# Machine Learning Assisted Experimental Characterization of Bubble Dynamics in Gas–Solid Fluidized Beds

**DOI:** 10.1021/acs.iecr.4c00631

**Published:** 2024-05-01

**Authors:** Shuxian Jiang, Kaiqiao Wu, Victor Francia, Yi Ouyang, Marc-Olivier Coppens

**Affiliations:** † Centre for Nature-Inspired Engineering and Department of Chemical Engineering, University College London, London WC1E 6BT, United Kingdom; ‡ School of Engineering and Physical Sciences, Heriot-Watt University, Edinburgh EH14 4AS, United Kingdom; § Laboratory for Chemical Technology, Ghent University, Ghent 9052, Belgium; ∥ Department of Chemical Engineering, Guangdong University of Technology, Guangzhou 510006, China

## Abstract

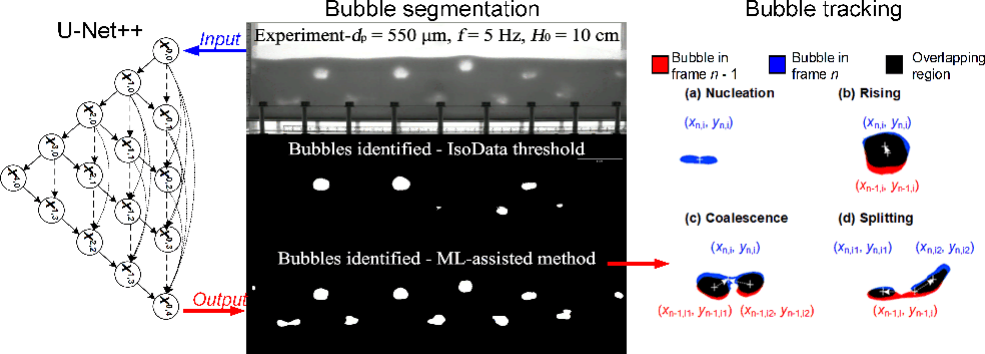

This study introduces a machine learning (ML)-assisted image segmentation method for automatic bubble identification in gas–solid quasi-2D fluidized beds, offering enhanced accuracy in bubble recognition. Binary images are segmented by the ML method, and an in-house Lagrangian tracking technique is developed to track bubble evolution. The ML-assisted segmentation method requires few training data, achieves an accuracy of 98.75%, and allows for filtering out common sources of uncertainty in hydrodynamics, such as varying illumination conditions and out-of-focus regions, thus providing an efficient tool to study bubbling in a standard, consistent, and repeatable manner. In this work, the ML-assisted methodology is tested in a particularly challenging case: structured oscillating fluidized beds, where the spatial and time evolution of the bubble position, velocity, and shape are characteristics of the nucleation-propagation-rupture cycle. The new method is validated across various operational conditions and particle sizes, demonstrating versatility and effectiveness. It shows the ability to capture challenging bubbling dynamics and subtle changes in velocity and size distributions observed in beds of varying particle size. New characteristic features of oscillating beds are identified, including the effect of frequency and particle size on the bubble morphology, aspect, and shape factors and their relationship with the stability of the flow, quantified through the rate of coalescence and splitting events. This type of combination of classic analysis with the application of the ML assisted techniques provides a powerful tool to improve standardization and address the reproducibility of hydrodynamic studies, with the potential to be extended from gas–solid fluidization to other multiphase flow systems.

## 1 Introduction

Hydrodynamics plays a key role in the operation and application of fluidized beds.^1^ Bubbles significantly alter their shape and size in any fluidized bed, from nucleation to growth and rupture and through coalescence and splitting. Capturing these changes experimentally is challenging, particularly for small bubbles. This type of uncertainty is especially true in complex bubbling systems, such as those in vibrating beds, oscillating units, or dynamically structured flows, where the evolution of the bubble shape depends on external actuation. A reliable, repeatable, and transportable methodology to measure bubble characteristics is important to monitor fluidization, optimize the bed, and provide direct comparison across different experimental setups and units. Depending on the nature and position of the sensors used, bubble measurement techniques can be broadly classified into two categories: (1) intrusive methods, such as pressure transducer, capacitive probes, and optical fiber analysis, where the probe could disturb the bubble behavior;^2^ and (2) nonintrusive methods, which include optical imaging,^3^ electrical capacitance tomography (ECT),^4,5^ magnetic resonance imaging (MRI),^6^ X-ray digital radiography (XDR),^7^ and X-ray computed tomography (XCT).^8,9^

Direct optical photography, followed by digital image analysis (DIA), is one of the simplest but also most widely used techniques to study the behavior and motion of bubbles in fluidized beds. DIA is particularly effective for analyzing quasi-2D fluidized beds due to the ease of operation, high accuracy, and capacity to visualize irregular boundary shapes and measure the size and velocity of bubbles. DIA can be used to remove noise from images as well as to enhance, restore, segment, and extract features. Therefore, it is widely used to analyze the shapes of bubbles in fluidized beds based on light transmission. Busciglio et al.^10^ developed a DIA technique to study bubble dynamics in a 2D fluidized bed. They tested two kinds of velocimetry techniques: (i) a Eulerian Velocimetry Technique (EVT), based on cross-correlation between subsequent frames, and (ii) a Lagrangian Velocimetry Technique (LVT), which tracks bubbles to monitor their evolution along the bed. Li et al.^11^ used DIA to assess the impact of pulsed gas frequencies on bed expansion, bubble diameter, bubble deformation, and bubble-rise velocity in a 2D fluidized bed, and they proposed a rising velocity model for fluctuating conditions.

However, DIA still has certain shortcomings that impact its efficacy, including difficulties in recognizing overlapping, out-of-focus, and overexposed bubbles. DIA is highly dependent on the quality of the image, local spatial characteristics such as grayscale and texture, and statistical uniformity of pixel properties. Achieving perfectly uniform illumination is often impractical,^12^ with conditions varying across experiments, studies and set-ups. To improve the standard of measurement and create a reproducible, transportable methodology, some researchers are exploring the potential of Artificial Intelligence (AI) and Machine Learning (ML) as alternative tools for bubble identification and segmentation. Applying ML techniques, recent studies have significantly advanced bubble recognition and tracking approaches in gas–liquid systems, outperforming traditional image analysis methods.^13^ Poletaev et al.^14^ developed a multistep neural network (NN) approach capable of identifying overlapping, blurred, and nonspherical bubbles in turbulent bubbly jets, achieving detection across volume gas fractions of 0 to 2.5%, only with errors up to 20% occurring at the edge of the measurement domain. Wang et al.^15^ coupled a convolutional neural network (CNN) with an improved three-frame difference method and an intersection-over-union (IoU) postscreening algorithm to extract bubble patterns in plate heat exchangers. This approach not only precisely captured and tracked individual bubble behavior and hydrodynamic events but also achieved an average precision rate of over 94%, significantly enhancing the bubble flow analysis. Furthermore, Seong et al.^16^ utilized a U-Net-based CNN model to segment bubbles during the boiling process in subcooled flow conditions, identifying bubble trajectories with more than 90% accuracy.

Bubble characteristics in gas–solid systems are distinct from those in gas–liquid systems, and bubble dynamics exhibits increased complexity. Unlike the mostly rounded bubbles shaped by surface tension in liquids, bubbles in gas–solid systems often assume irregular forms, further complicating tracking. Instability is another challenge, with particles potentially raining from the roof of bubbles, causing bubbles to fragment into multiple parts. While conventional image analysis tools struggle to address these challenges effectively, machine learning methods have seen increasing use in various aspects of the investigation of gas–solid fluidized beds, such as optimizing image reconstruction,^17^ accelerating simulations,^18^ and discovering correlations between properties.^19^ Despite this progress, the literature on applying ML techniques specifically to image segmentation for bubble identification and behavior analysis in gas–solid fluidized beds remains sparse. Fu et al.^20^ implemented deep learning techniques, specifically using the DeepLab V3+ model, for bubble segmentation in the automatic identification of bubbles within a gas–solid bubbling fluidized bed. Their application of this advanced model yielded highly accurate results, demonstrating a remarkable segmentation accuracy of 97.95%.

Oscillating fluidized beds provide a well-controlled yet particularly challenging benchmark for work in a standard methodology for bubble image analysis and tracking. They are applied in various industrial processes, including drying, combustion, and coal separation.^21,22^ The introduction of oscillatory flow significantly alters the behavior of both bubbles and particles. Pulsation effectively reduces the number of bubbles, improves fluidization quality, and the mass and heat transfer efficiency by suppressing channel flow and short-circuiting and enhancing the particle separation efficiency.^23−25^ The bubbling dynamics are the main driver of the system performance, affecting key operational features such as gas and particle residence and contact time, particle entrainment, and reaction conversion.^26^ Many works have studied this relationship experimentally and computationally since the early work of Wong and Bair, who conducted experiments on a pulsed cylindrical fluidized bed operating across a range of frequencies (1–10 Hz). Wong and Bair utilized a piston model to calculate the natural frequency of the fluidized bed, and concluded that the most pronounced effects of pulsation occur when the applied frequency aligns with the bed natural frequency.^27^ Among many others, computational fluid dynamics (CFD) research, conducted by Wang and Rhodes,^28^ employed the discrete element method (DEM) to simulate fluidization of a quasi-2D, rectangular bed of spherical Geldart-B particles subjected to both square-wave and sinusoidal pulsed flows to emulate experimental conditions studied by Coppens et al.^29^ Their work revealed that higher amplitudes led to more distinct horizontal channel-like structures near the gas distributor, more frequent bubble coalescence, and greater instability. Setting the offset of the oscillating flow to the minimum fluidization velocity led to a more stable structured flow. However, the stability of computational structured bubbling flows to date is not as high as in the patterns observed experimentally by Coppens et al.,^29^ who have shown that an oscillatory gas flow can effectively rearrange bubbles into a stable triangular lattice with controlled wavelength and bubble size, irrespective of the bed width. Further experimental and computational work in our group has studied the bubbling dynamic under various pulsation conditions in rectangular and annular quasi-2D fluidized beds.^29−31^ The features of these patterns, called *dynamically structured fluidized beds*, are driven by both the gas pulsation conditions (frequency, amplitude, and offset) as well as the properties of the solids in the bed.

In this work, we introduce an ML-assisted methodology to improve the characterization of bubble flows and their dynamics, and we report a comprehensive case study applying this new methodology in the context of structured oscillating fluidized beds. The segmented images obtained through the ML method are used by an in-house Lagrangian algorithm to identify and track bubbles and to compute the evolution of key properties and events over time in a quasi-2D fluidized bed. The approach presented here is fully automatic and is shown to offer a high degree of robustness across beds of different illumination, particle sizes and varying bubble sizes, velocities, and degrees of coalescence and splitting, all of which are crucial metrics for understanding the hydrodynamics of any fluidized bed.

## 2 Experiments

**Figure 1:**
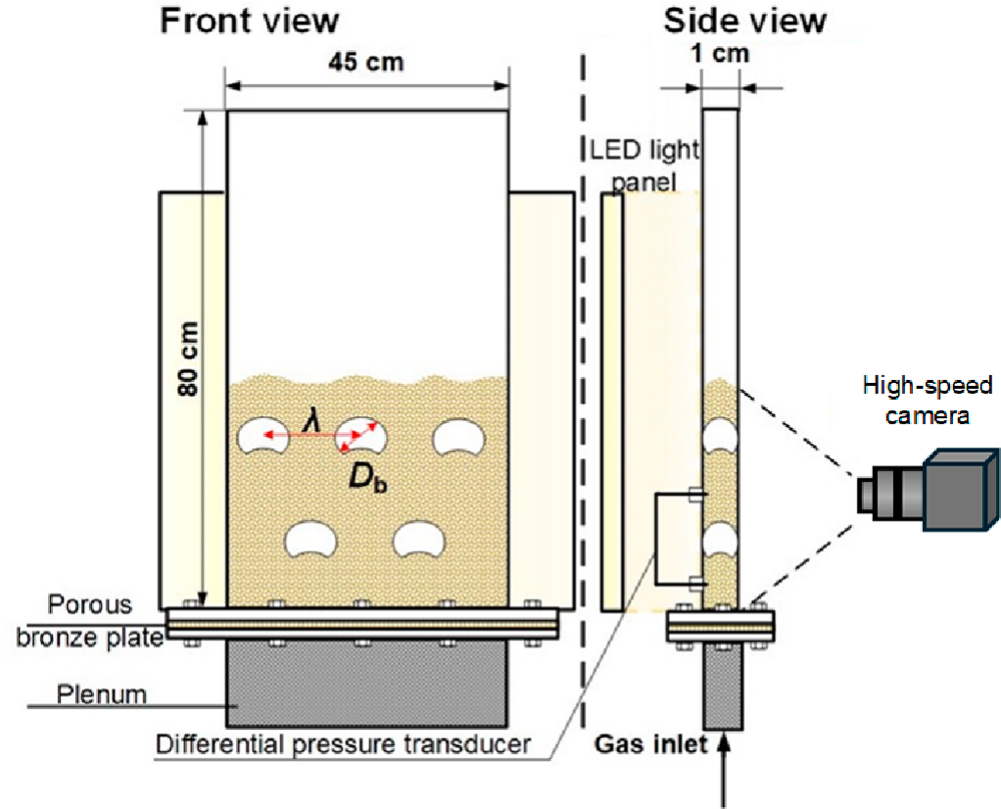
Schematic diagram of the quasi-2D fluidized bed setup with a pulsed gas flow.

A quasi-2D pulsed fluidized bed is used to visualize the gas–solid fluidization process. Figure 1 shows the experimental setup, consisting of a fluidized chamber made of Plexiglas, a gas distributor, and a plenum chamber filled with material to facilitate a uniform gas distribution. The quasi-2D bed section is 80 cm high, with a cross-section of 45 cm × 1 cm. A porous bronze plate (Grade 07, BK 10.30.07, Sintertech) with a thickness of 3 mm acts as the gas distributor. To provide background lighting and adjust the contrast between bubble phase and dense phase, a square 595 mm × 595 mm LED backlit panel (ROBUS, 40W) was used, placed directly behind the fluidized bed. A Basler digital camera (acA1920–150um) equipped with a wide-angle lens (MachineVision M1224-MPW2) was used to capture the regime transitions with a pixel resolution of 2592 × 1000 at 100 fps.

The oscillatory inlet gas flow is created via two branches, combining a steady and a pulsed flow. The latter is created by an MKS 154B type proportional solenoid valve, which produces a sinusoidal oscillation in the gas flow rate. Gas flow rates in the two branches were measured using two well-calibrated mass flow meters (Omega FMA-1611A). A differential pressure transducer (Omega PX409) was mounted on the rear wall of the fluidized bed. The solenoid valve, pressure sensor, and flow meters were all connected to a data acquisition board (National Instruments USB-6211). This setup allowed for the control of the solenoid valve and the recording of pressure and flow rate signals through a LabVIEW program (National Instruments, Version 2019, sampling frequency = 1000 Hz). Carefully sieved glass beads with a density of ρ_p_ = 2500 kg·m^–3^ were used as the bed material. As shown in Table 1, particles with different average sizes, falling into the Geldart B group, were used in the experiments. The normalized inlet oscillatory flow velocity *û* is defined as
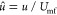
1
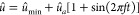
2where *u* is the inlet gas flow velocity, *U*_mf_ is the minimum fluidization velocity. *û*_a_ and *û*_min_ are the normalized amplitude and minimum velocity of the applied oscillatory flow, respectively, each normalized by *U*_mf_. *f* denotes the oscillating flow frequency. Experiments were conducted under different conditions, as shown in Table 1. For each condition, the bubble behaviors were recorded for 10 s, resulting in a total of 1000 frames of bubble images; pressure drop and flow rate were recorded for 1 min.

**Table 1:** Experimental Conditions

	Particle diameter, *d*_p_ (μm)			
	238	375	475	550
Minimum fluidization velocity, *U*_mf_ (cm/s)	4.58	11.05	17.09	22.1
Amplitude, *û*_a_	1.50	0.42	0.36	0.30
Offset, *û*_min_	1.0	1.02	1.03	0.92
Initial bed height, *H*_0_ (cm)	10			
Frequency, *f* (Hz)	3–7			

## 3 Data Analysis

### 3.1 Bubble Segmentation

This section outlines the challenges associated with bubble recognition and introduces the U-Net-based convolutional neural network architecture. Subsequently, the bubble segmentation framework is presented, including the imaging preprocessing module and the model training process.

#### 3.1.1 Challenges in Bubble Segmentation

After the flow pattern of a quasi-2D fluidized bed was recorded, the bubble phase could be separated from the emulsion phase using image processing techniques. Currently, DIA of bubble flow captured by high-speed camera mainly relies on the gray level in each frame to identify the bubble phase and calculate bubble size and shape.^32,33^ In our previous works, a DIA procedure was employed to convert the recorded images into a serious of binary images, which were then used to identify the bubbles with the particle analysis module in ImageJ v1.52a.^30,34^ A specific threshold value for pixel intensity, calculated based on the ISODATA algorithm,^35^ was required to distinguish between the bubble or dense phase and obtain the binary images. To ensure the correct threshold was applied, visual inspections were conducted to verify the phase separation.^36^

**Figure 2:**
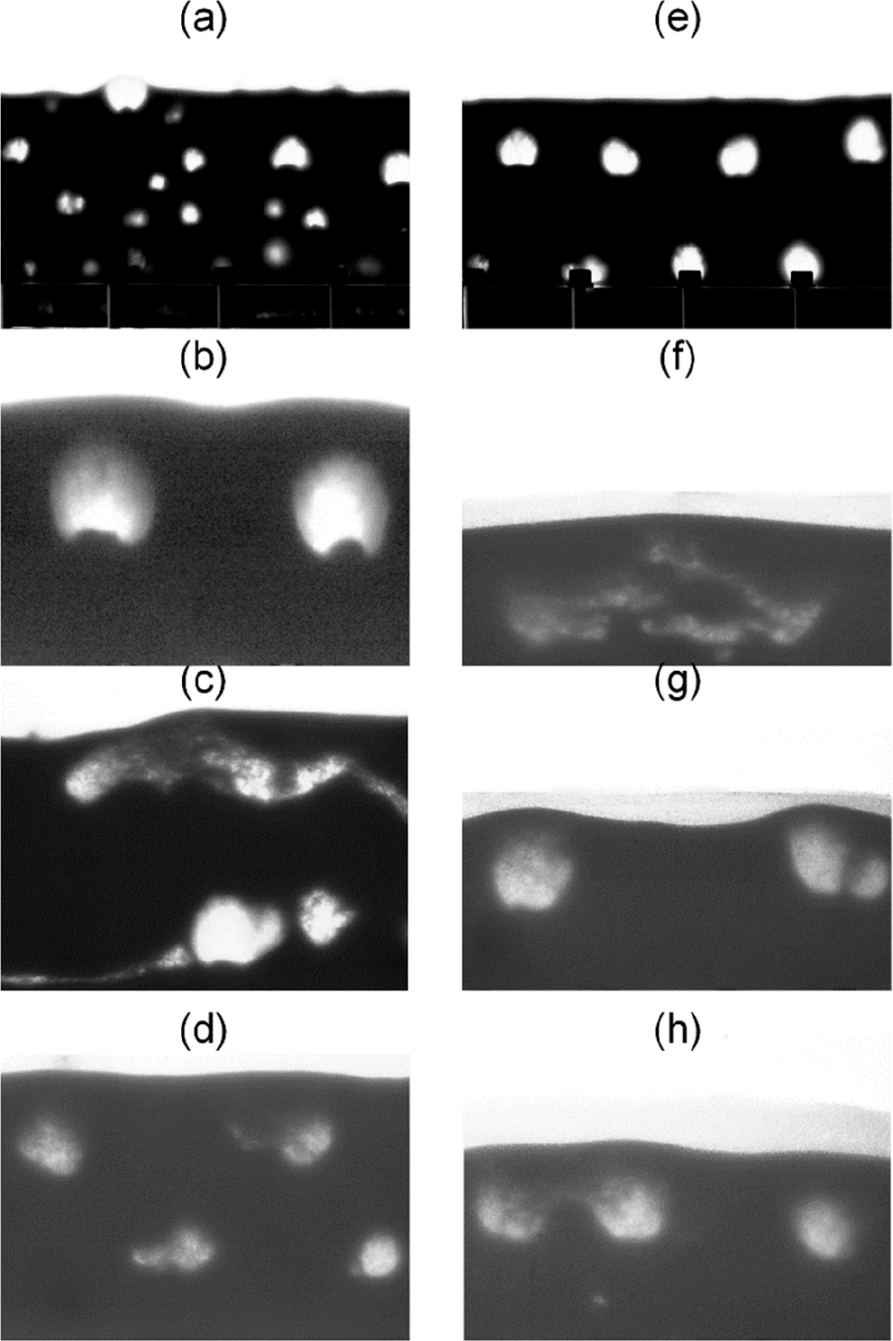
Examples of bubble or void images in a quasi-2D fluidized bed with a bed height of *H*_0_ = 10 cm. Examples of well-lit natural (a) and dynamically structured beds (e) and poorer quality images of oscillating beds of (b) *d*_p_ = 238 μm, *f* = 5 Hz; (c) *d*_p_ = 375 μm, *f* = 5 Hz; (d) *d*_p_ = 475 μm, *f* = 5 Hz; (e) structured bed; (f) *d*_p_ = 550 μm, *f* = 3 Hz; (g) *d*_p_ = 550 μm, *f* = 5 Hz; (h) *d*_p_ = 550 μm, *f* = 7 Hz.

Figures 2a and 2e illustrate the differences between the chaotic bubbling dynamics characteristic of a natural bed, as shown in Figure 2a, and a dynamically structured bed, as shown in Figure 2e, where bubbles arrange in a triangular lattice. Accurate identification of bubble properties such as location, size, and shape is crucial for determining the stability of such bubble patterns. Figures 2a and 2e showcase examples of well-lit images with good focus, definition, and contrast, while the remaining panels present examples with poorer illumination and oscillating beds with larger particle diameters and more complex dynamics that render bubbles with blurred boundaries and irregular shapes. Establishing a precise threshold to properly segment the bubble phase in these cases poses a significant challenge and introduces uncertainty, particularly in the metrics assigned to smaller bubbles with poor contrast. The pixel intensity within the bubbles displays an uneven distribution, further complicating the segmentation process. Furthermore, when glass beads are used in quasi-2D experimental deposits often build up in the chamber walls due to electrostatic forces. In these experiments, a layer of particles tends to adhere to the walls because of triboelectric charging. As the thickness of this layer equals the particle size, when the particle size increases, it further blurs the boundary between the bubbles and the dense phase.

#### 3.1.2 Architecture of U-Net

**Figure 3:**
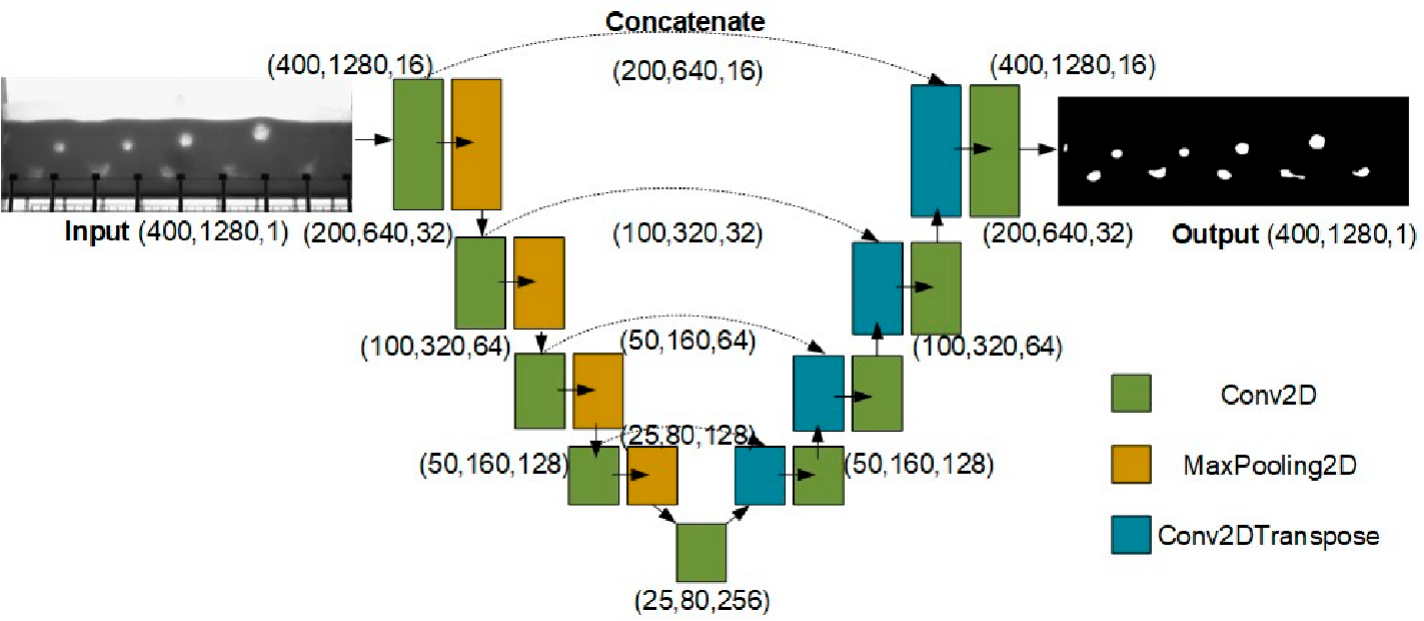
Example of the U-Net architecture used in this study. Input image sizes are 400 × 1280 pixels.

To offer a more systematic, faster, and more reproducible method for bubble identification, a machine learning (ML)-assisted approach is proposed. This approach transforms the grayscale image into a binary image, effectively removing the need for a threshold. Consequently, this methodology is well-suited to handling situations with nonuniform lighting conditions and complex backgrounds, thus improving the reproducibility of direct comparisons across experimental setups. The method is based on a U-Net segmentation network, which is commonly used in semantic segmentation due to its efficiency with fewer training data and fewer classes.^37^ Although initially developed for cell detection, U-Net has been found to be easily adaptable for the detection and segmentation of arbitrary structures involving more complex objects. As shown in Figure 3, U-Net adopts an encoder-decoder neural network design derived from the fully convolutional neural networks architecture.^37^ The encoder extracts the depth features through the convolution operation, described by Conv2D layers, and after each Conv2D layer, a MaxPooling2D operation downsamples the feature maps to capture larger contextual information. The decoder performs the upsampling operation to recover the spatial resolution through Conv2DTranspose layers, effectively performing the inverse of Conv2D, and fuses the feature maps of the same size into the shallow layer. Along with transfer learning, the U-Net architecture has proven effective for segmenting bubbles or particles from diverse backgrounds with the help of very few additional ground truth segmented images that act as training data.^16,38,39^

#### 3.1.3 Workflow

**Figure 4:**
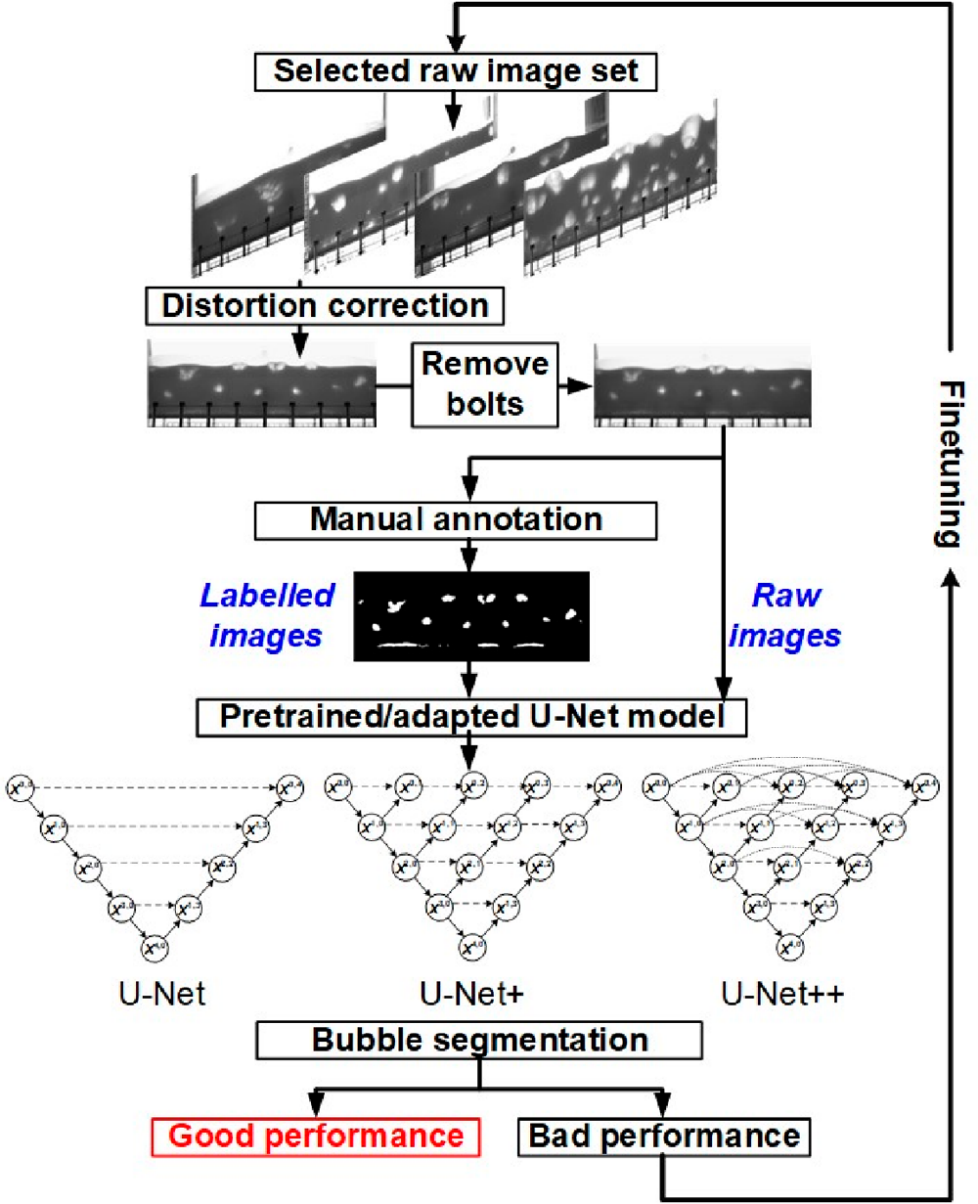
Bubble segmentation model development.

The process followed to develop the automated bubble segmentation tool is depicted in Figure 4. First, to generate a training set for image analysis, data sets were prepared in the form of raw images (experimental flow images) and labeled images (ground truth images). Original images under different conditions were selected. The image preprocessing in this study was carried out using the Python library OpenCV (version 4.8.0). A slight barrel distortion can be observed in the images. To correct this, a global correction method applying pincushion distortion with appropriate distortion parameters was applied to every raw image. The bolts above the gas distributor were removed from the images by the TELEA inpainting algorithm.^40^ A set of 120 ground truth images was obtained through manual processing, and Adobe Photoshop was used to draw the bubble boundaries in the sampling images. The manually segmented images were performed subjected to a 5 × 5 Gaussian blur and binary thresholding to enhance accuracy and smoothen the binary representations, particularly focusing on refining bubble edges. The background and dense phase in the images correspond to black, and the bubble phase is represented in white.

The next step involves constructing and training the U-Net. U-Net+ and U-Net++ are improved models from U-Net, designed to alleviate the issue of unknown network depth by employing an efficient ensemble of U-Nets of varying depths. These models partially share an encoder and colearn simultaneously using deep supervision.^41^ U-Net+ and U-Net++ were also trained and tested in this work. A total set of images, resized to a pixel size of 1280 × 400, was used. Weighted binary cross entropy is adopted as the loss function,^42^
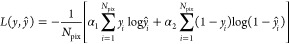
3where subscript *i* denotes the pixel *i*, *y*_i_ and *ŷ*_*i*_ represent the pixel in the binary mask, and its probability score computed by the sigmoid activation layer, respectively. *N*_pix_ represents the number of pixels in the binary image,  and  are weight parameters, and *x* and *x* are the numbers of nonedged and edged pixels in the binary mask, respectively. The Adam optimizer was employed with a learning rate of 0.0003 for 200 epochs,^43^ after no further improvement in the training and validation loss was observed. Checkpoints were saved with the highest mean Dice score achieved on the validation set during training. Bubble diameters and distributions were calculated by utilizing Python libraries NumPy, math, and pandas.

**Figure 5:**
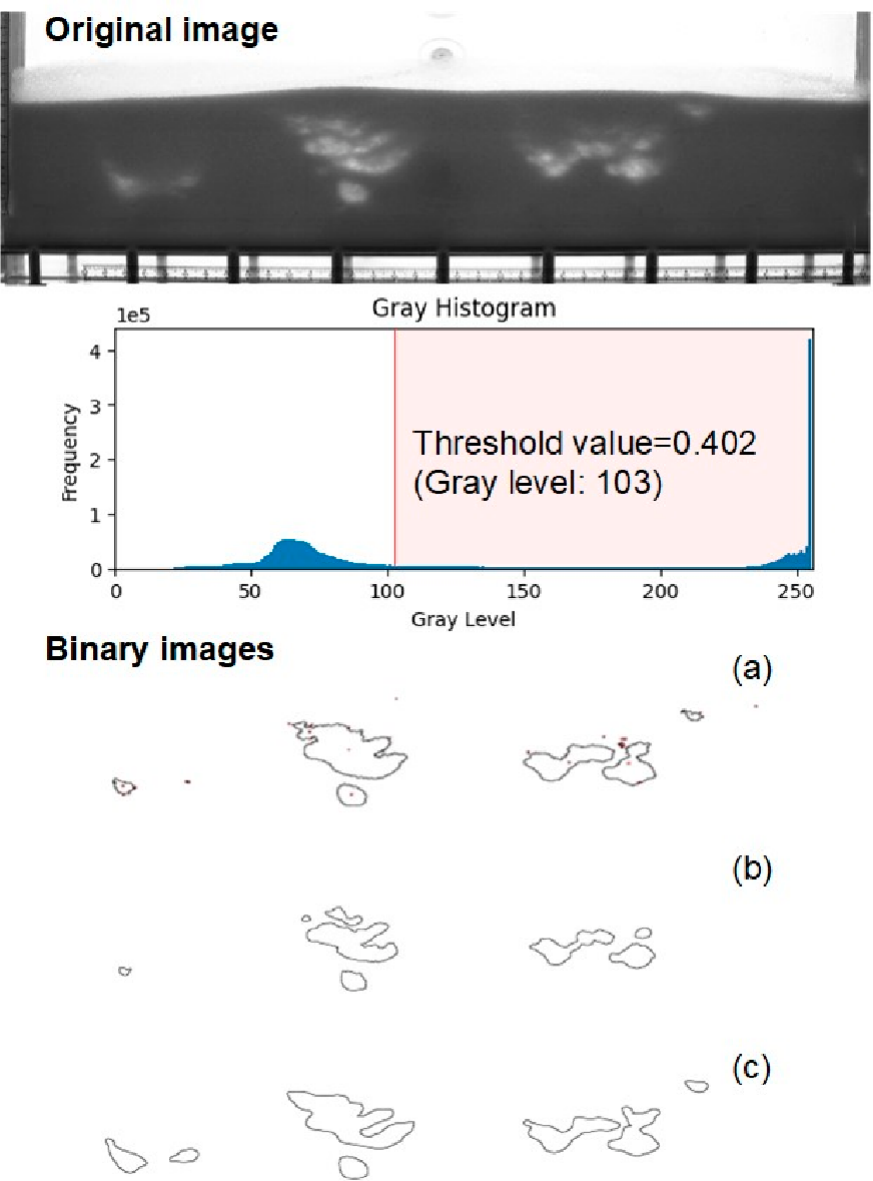
Example of original image with the gray level histogram and corresponding recognized bubble images generated by (a) ISODATA threshold; (b) image independent threshold; and (c) U-Net segmentation.

In Figure 5, images (a) and (b) are processed through the DIA technique. Both involve determining a threshold for binarizing images by analyzing the statistics of the pixel intensities. In ISODATA thresholding, pixels are assigned to two classes: background and object. The mean intensities of these two classes are calculated, and the threshold is updated and determined based on these values. In the second thresholding method, an image-independent threshold, denoted as *T* = 0.9 ⟨*I*⟩ is applied, where ⟨*I*⟩ is the average image intensity.^36^ Comparing the three binary images obtained through different methods, the bubbles acquired using the U-Net method (Figure 5c) exhibit a closer resemblance to those observed in the raw image, demonstrating an enhanced capability to accurately track the bubble’s shape. More specifically, the binarized bubble images from both DIA methods lose some information, notably for bubbles located on the left side of the image, which exhibit a lower contrast and more blurred boundaries.

### 3.2 Bubble Tracking and Velocity Measurement

Tracking the motion of the bubbles over time is crucial for computing bubble split-up and coalescence statistics, which determine the evolution of bubble size distributions during fluidization. A Lagrangian Velocimetry Technique is employed to track bubbles, wherein each bubble is assigned a unique ID, facilitating the tracking of individual bubbles across time frames.

**Figure 6:**
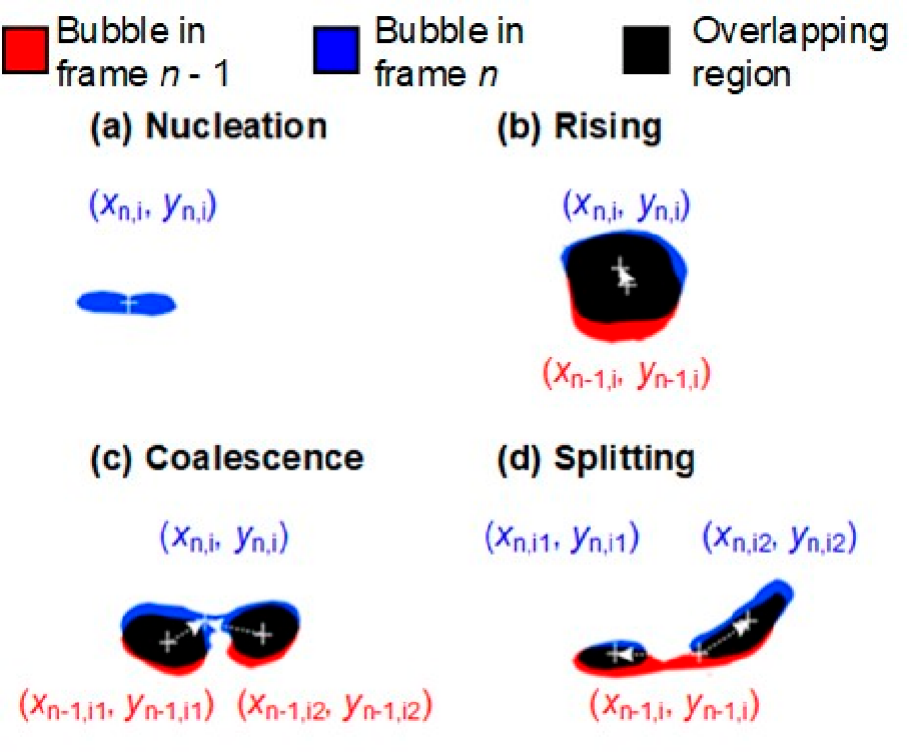
Examples for bubble matching cases: (a) No matching, bubble nucleation in the current frame. (b) One-to-one matching, bubble rising in the current frame. (c) Multi-to-one matching, bubble coalescence. (d) One-to-multi matching, bubble splitting.

As illustrated in Figure 6, the tracking procedure for different individual cases can be described as follows:

1. Bubble nucleation: When no shared overlapping region can be identified in frame *n* (Figure 6a), recognize the area as a new bubble nucleated in frame *n* – 1.
2. Bubble rising: When only one overlapping region can be identified with the previous frame *n* – 1 (Figure 6b), and the bubble diameter and centroids in previous frame *n* – 1 and current frame *n* meet the following conditions:
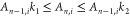
4where *k*_1_ and *k*_2_ are two constant factors, with *k*_1_ = 1/*k*_2_.
3. Bubble coalescence: When in the current frame *n*, multiple parent bubbles in the previous frame *n* – 1 overlap with one daughter bubble (Figure 6c), and meet the condition:
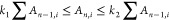
5
4. Bubble splitting: When in current frame *n*, a single parent bubble in the previous frame overlaps with multiple daughter bubbles (Figure 6d), meeting the condition:
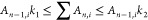
6

**Figure 7:**
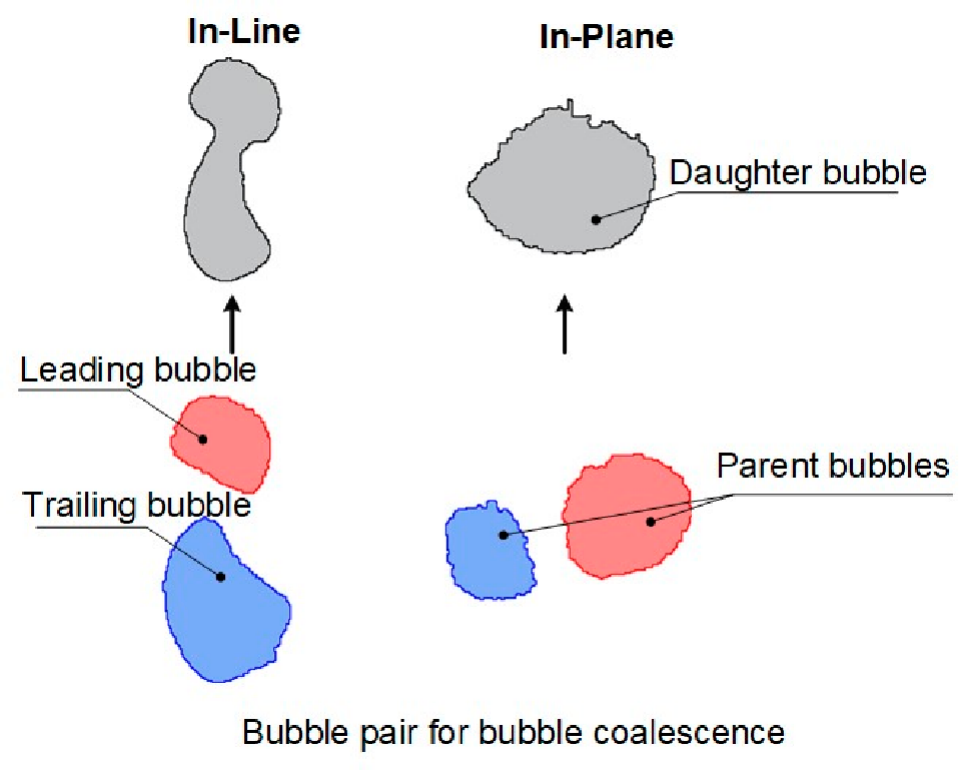
Bubble coalescence types in a fluidized bed.

Bubble coalescence in bubbling fluidized beds can be classified into two types: in-line and in-plane (Figure 7). In-line coalescence occurs between two vertically aligned rising bubbles. The trailing bubble accelerates upon entering the wake of the leading bubble and subsequently merging with it. In-plane coalescence, on the other hand, takes place between horizontally aligned adjacent bubbles, where bubbles coalesce laterally.^44,45^ For in-plane coalescence, two adjacent bubbles at nearly identical vertical positions merge as they ascend along their trajectories.

For the measurement of the bubble velocity, the binary image is first processed to filter out and remove horizontal void channels with a shape factor φ < 0.25 and bubble aspect ratio β > 10 (see eq 10 and eq 11). Subsequently, very small bubbles with a size smaller than 10 pixels × 10 pixels are also eliminated. Given that the image capturing frequency is sufficiently high, *k*_1_ and *k*_2_ were adopted to account for bubble shrinkage and expansion in bubble area: *k*_1_ = 0.64 and *k*_2_ = 1.5625, respectively. A comprehensive explanation of how the values for *k*_1_ and *k*_2_ were determined is detailed in the Supporting Information. Once all bubbles are indexed and an indexed bubble with the same subscript *i* appears in two subsequent frames, *n* – 1 and *n*, the displacement is computed as the difference of the bubble centroid coordinates:
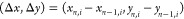
7

For the rising bubbles and daughter bubbles that split in current frame *n*, the velocity is calculated as follows:
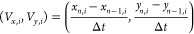
8

For the daughter bubbles from coalescence in the current frame *n*, the velocity is calculated by
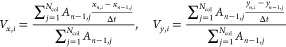
9where *j* represents the parent bubbles that generate daughter bubble *i*, and *N*_col_ denotes the number of parent bubbles.

### 3.3 Bubble Properties

The equivalent bubble diameter *D*_b_ was determined by eq 10:
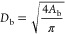
10where *A*_b_ refers to the segmented bubble area. Bubble deformation can be characterized by the bubble shape factor φ and bubble aspect ratio β. φ and βare defined as follows:
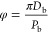
11
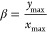
12where *P*_b_ is the perimeter of the bubble; *y*_max_ and *x*_max_ denote the maximum length of the bubble in the vertical and horizontal directions, respectively. For a circular shaped bubble, 0.9 < β < 1.1, for an elliptical or cap shaped bubble, β < 0.9, and for an elongated bubble, β > 1.1.^11^

The bubble centroid ***c*** is calculated by the mean of all the pixels within the bubble under investigation:
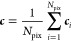
13where *N*_pix_ is the number of the pixels and ***c***_i_ is the position of pixel *i*.

## 4 Results and Discussion

### 4.1 Network Comparisons and Validations

**Figure 8:**
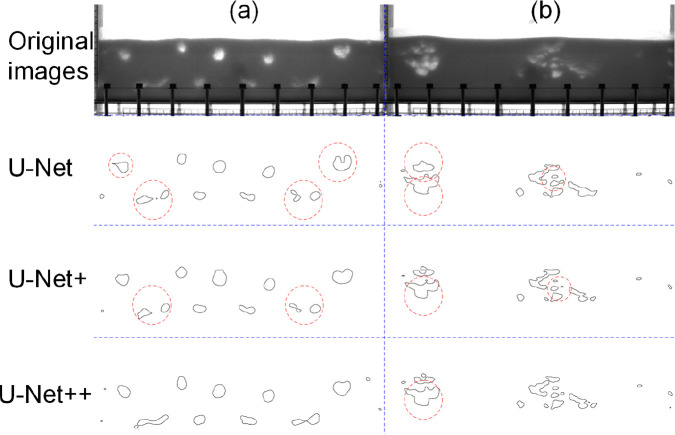
Results of bubble segmentation using different U-Net methods: (a) *d*_p_ = 550 μm, *f* = 5 Hz. (b) *d*_p_ = 475 μm, *f* = 3 Hz.

To assess the performance of U-Net across the different architectural designs mentioned in Figure 4, Figure 8 presents sample images of bubble segmentation results, including two cases involving significantly different bubble morphologies. Bubbles that are either not fully segmented or partially segmented are highlighted with red circles. Comparing the areas marked, it is clear that U-Net++ achieves the best bubble segmentation performance. U-Net++ was selected and applied in all subsequent analyses.

In order to quantitatively assess the performance of bubble segmentation across different methods, we utilized Acc (Pixel Accuracy), F1-score, and IoU (Intersection over Union), which are widely used in segmentation methods.^46^ Acc quantifies the proportion of pixels correctly classified in the predicted segmentation versus the ground truth. The F1-score acts as a balanced measure between precision and recall for binary segmentation tasks, offering a harmonic mean of the two. IoU assesses the overlap between the predicted segmentation and the ground truth, serving as a direct measure of segmentation accuracy.

The evaluation encompassed 30 test data sets from fluidized beds with *d*_p_ = 550 μm and *f* = 0, 3, and 5 Hz. These conditions, as shown in Figure 2, present a significant challenge for bubble segmentation and identification. The results summarized in Table 2 demonstrate the superior performance of U-Net++ across all indicators over all other methods tested. Notably, all U-Net methods significantly outperform the ISODATA thresholding approach, highlighting the advanced capabilities of these neural network models in handling complex segmentation tasks.

**Table 2:** Comparison of Bubble Segmentation Methods Using Different Scoring Techniques^a^

Methods	Acc (%)	F1-score (%)	IoU (%)
ISODATA threshold	97.2	10.72	5.66
U-Net	99.51	90.87	83.27
U-Net+	99.54	91.00	83.49
U-Net++	99.57	91.84	84.92

a Acc: pixel accuracy; F1-score; IoU: intersection over union.

**Figure 9:**
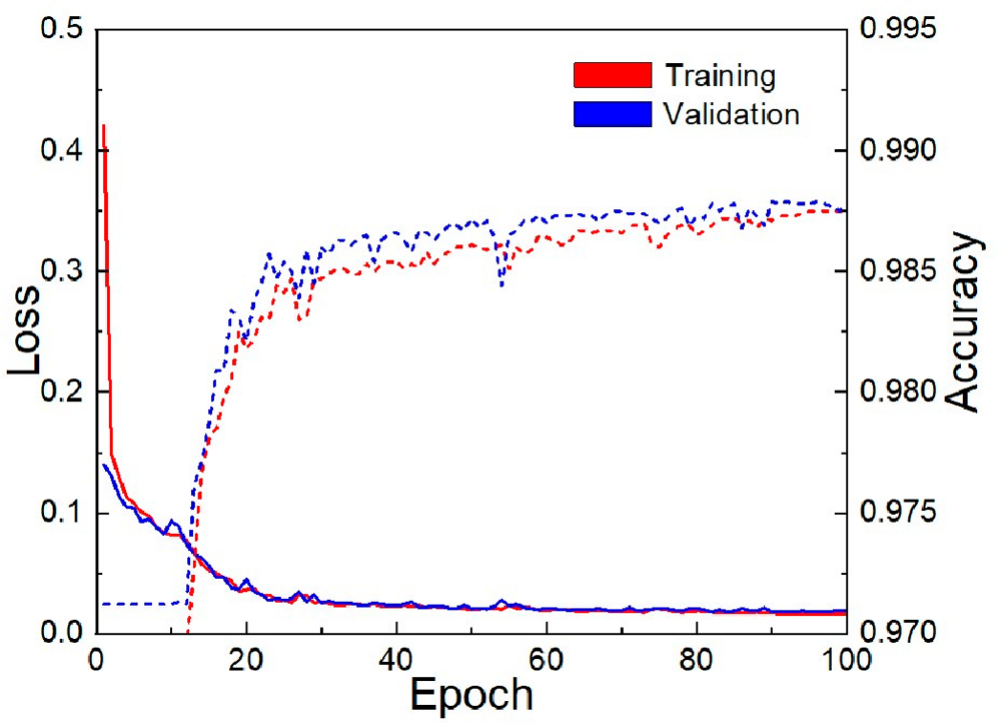
Loss and accuracy values of U-Net++ during the training and validation processes.

Figure 9 shows the evolution of the U-Net++ model accuracy and loss during the training and cross-validation processes throughout epochs. The training was stopped early after 100 epochs, as no further improvements were observed in either training or validation loss. The plotted accuracy and loss curves reveal a rapid convergence rate, with accuracy exceeding 98.75% and maintaining a low loss around 0.02. This highlights the effectiveness and efficiency of the model.

### 4.2 Individual Bubble Dynamics Analysis

**Figure 10:**
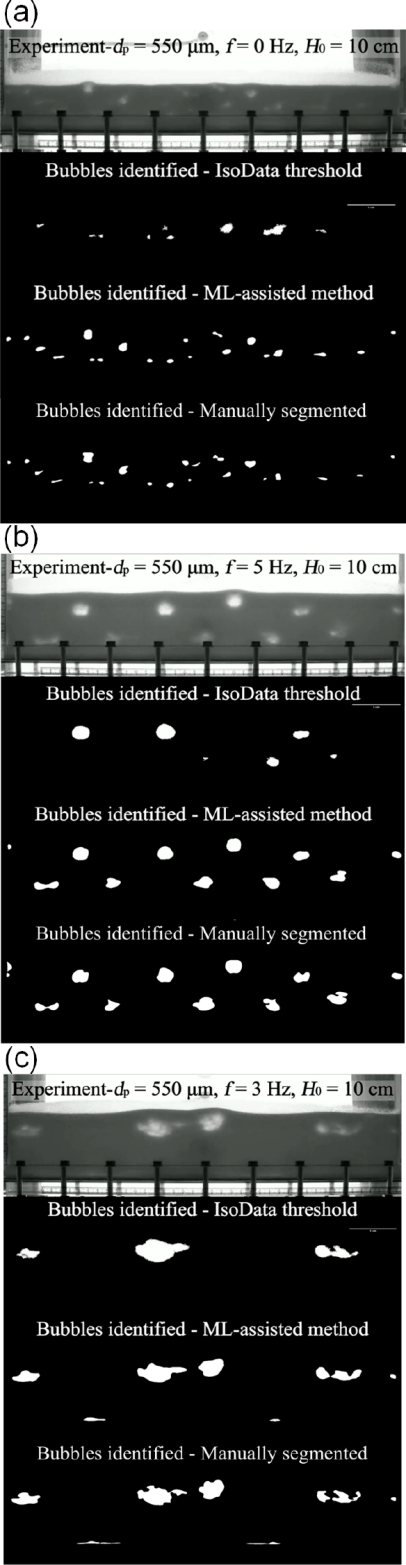
Examples between ISODATA threshold and ML assisted segmentation for (a) conventional beds and dynamically structured oscillating beds with (b) regular and (c) irregular bubble morphologies. Videos are available in Supporting Information.

The methodology described in Section 3 has been applied to study quasi-2D beds of different powders, under various flow conditions and illumination setups. The ML-assisted method is especially useful for identifying bubbles when the dynamics are complex, such as when they split, coalesce, or rupture. It is less sensitive to changes in illumination, areas out-of-focus, and the practical issues arising from obstructions to the view and deposits that blur the boundaries between the solid and the bubble phase. Figure 10 provides some examples comparing the segmentation in different beds, including a natural fluidized bed (a) and examples of oscillating beds where bubbles can be shaped regularly (b) or display complex changes in morphology (c). Overall, the ML-assisted method overall outperforms the traditional threshold method. It is able to capture bubbles during rupture (see Figure 10b) and after splitting (see Figure 10c), is unaffected by the presence of bolts at the bottom of the unit (see Figure 10b) and provides a more consistent measure of the bubble size, demonstrating effectiveness and versatility. Additionally, manually segmented bubbles in Figure 10 further validated the trained model’s capability in identifying and delineating bubbles over diverse cases.

Reducing uncertainty in segmentation and enabling the study of systems with low gas velocities and small bubbles facilitates the analysis and the standardization of hydrodynamic studies. It also provides detailed data, such as coalescence and splitting rates, which are otherwise hard to obtain. In the following sections, we illustrate the potential of this tool with a case study applying the ML-assisted method to characterize bubbling in oscillating beds made of differently sized particles and reporting a detailed analysis of the evolution of bubble size, velocity, morphology, and multibubble interaction phenomena.

#### 4.2.1 Bubble Rising Velocity

**Figure 11:**
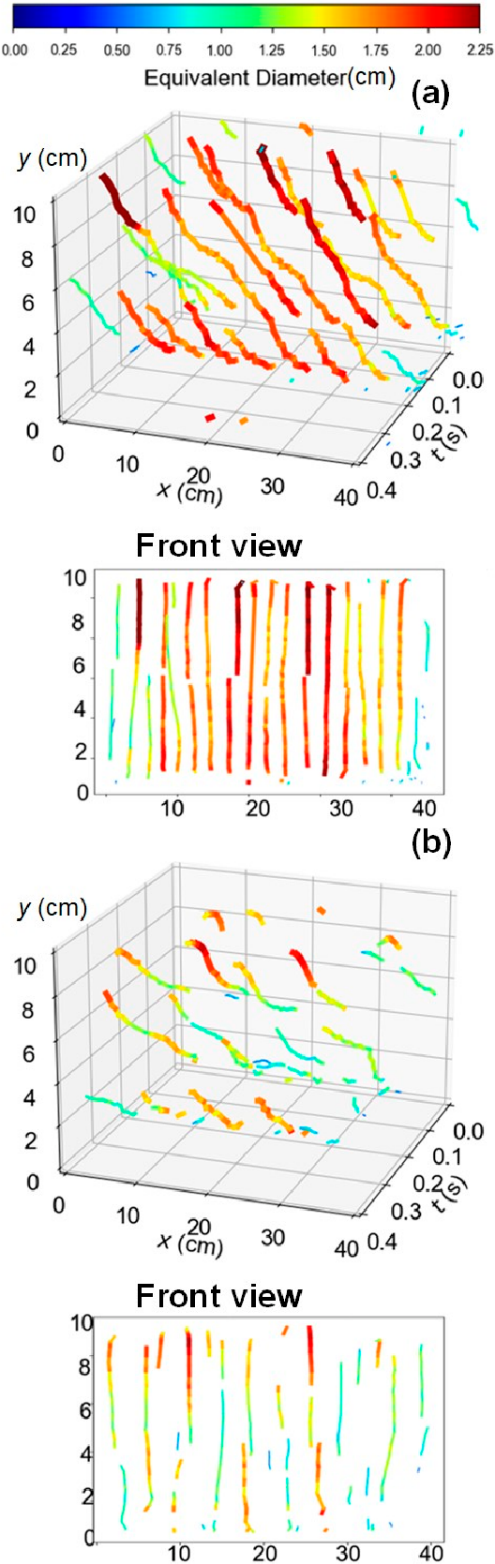
Bubble trajectories: each line refers to an individual bubble trajectory. Different line widths and colors represent the bubble equivalent diameter (*f* = 5 Hz, *H*_0_ = 10 cm). (a) *d*_p_ = 238 μm,  = 1.50,  = 1.0. (b) *d*_p_ = 550 μm,  = 0.30,  = 0.92.

The bubble rising velocity affects their residence time within the fluidized bed and the efficiency of the gas–solid contact. This section is devoted to specifically analyzing the dynamics of rising bubbles, excluding bubbles marked with nucleation, coalescence, and splitting events to concentrate on the rising motion of bubbles. To illustrate the algorithm, Figure 11 shows the distribution and evolution of bubble trajectories in a pulsed bed over two pulse periods (*f* = 5 Hz) for two representative cases with particles of 238 μm () and 550 μm , respectively. These two conditions have similar dimensionless amplitudes and close absolute offsets, and both cases render structured bubble flow.

Figure 11 displays only the rising motion of the bubbles, where the varying trajectory widths and colors correspond to different bubble diameters. As expected, in beds with particles of *d*_p_ = 238 μm, the bubble trajectories are more regular (Figure 11a), with bubbles rising in a straight line and without significant growth. All bubbles nucleate at fixed positions, as expected in a structured bed. The minor deviations from a straight line observed in the 2D representation are caused by the oscillatory flow. As the size of the particles increases in Figure 11b, the bed still develops a structured flow, where bubbles nucleate at fixed positions. A variation in bubble size is now noticeable along the trajectory, which shows more lateral movement, indicative of bubble breakage, coalescence, or rupture, and in general, a more unstable structured flow.

Darton et al.^47^ hypothesized that bubbles tend to rise along preferred pathways, and bubble rise velocity depends solely on bubble size. Davidson et al.^48^ proposed the following well-known relationship between bubble rising velocity and bubble diameter:
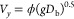
14

**Figure 12:**
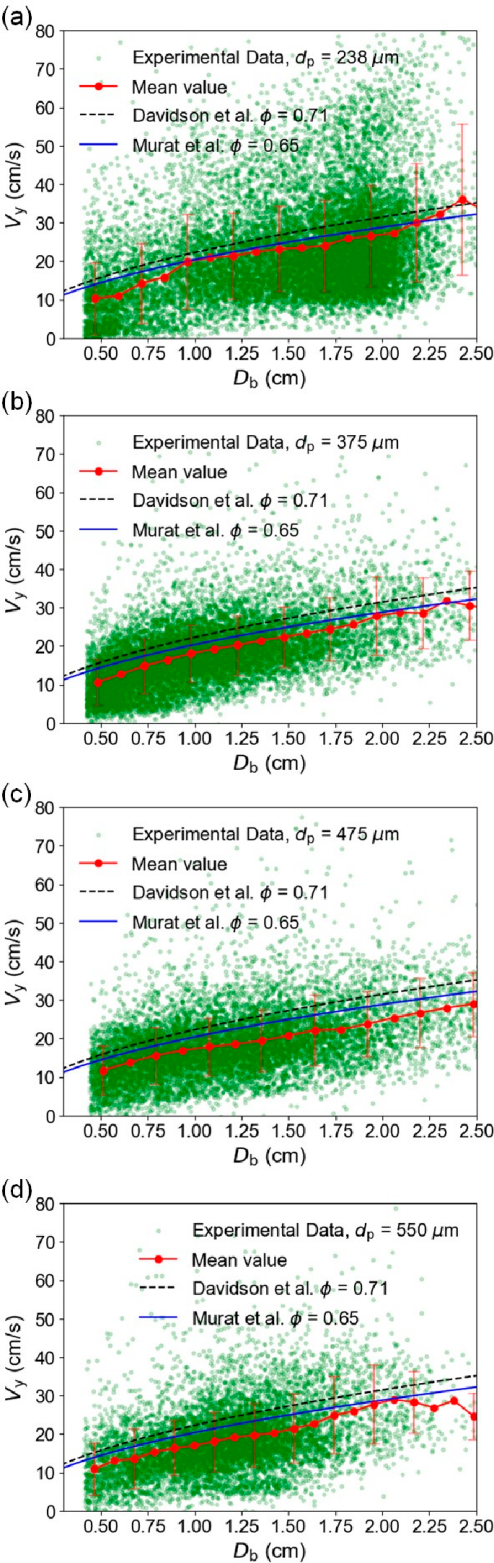
Comparison of bubble rising velocity versus equivalent bubble diameter with well-known correlations (*f* = 5 Hz, *H*_0_ = 10 cm): (a) *d*_p_ = 238 μm; (b) *d*_p_ = 375 μm; (c) *d*_p_ = 475 μm; (d) *d*_p_ = 550 μm.

The velocity coefficient ϕ is generally acknowledged to vary based on the properties of the particles involved.^49^ In the study by Davidson et al., ϕ is approximately 0.71 for Geldart B particles in 3D fluidized beds. For Geldart B particles in a pulsed quasi-2D fluidized bed, Murat et al.^50^ proposed that the coefficient takes the value ϕ = 0.64. In Figure 12, based on the experimental data, the correlations for predicting bubble rising velocity proposed by Davidson et al. and Murat et al. are plotted for comparison. Additionally, the mean experimental bubble rising velocities in relation to the equivalent bubble diameter are plotted. The vertical bars represent the standard deviation for each bubble diameter bin.

**Figure 13:**
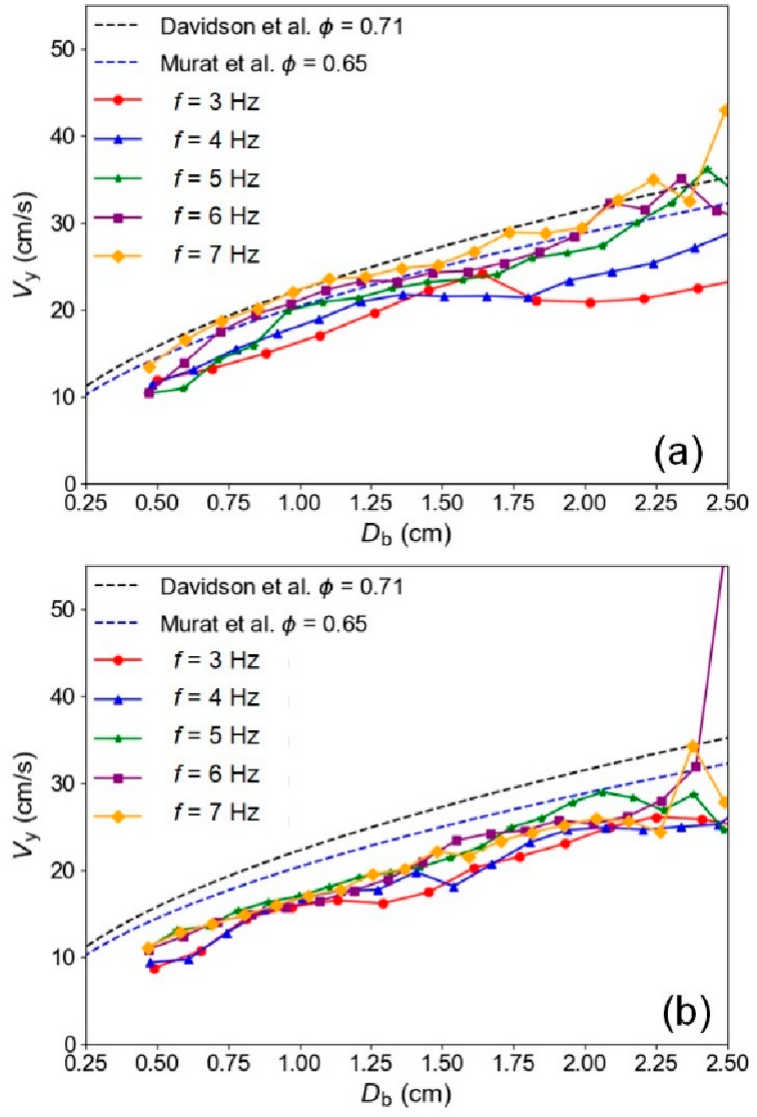
Effects of the pulsation frequency on the bubble rising velocity at different equivalent bubble diameters, for (a) *d*_p_ = 238 μm,  = 1.50,  = 1.0. (b) *d*_p_ = 550 μm,  = 0.30,  = 0.92. Error bars are excluded for clarity. Standard error ranges from 6.01 to 27.75 cm/s.

As indicated in Figure 12, at the same pulsation frequency, the bubble rising velocities are, on average, slightly lower than those expected in the literature for a natural fluidized bed. Furthermore, this deviation becomes increasingly significant as the particle size increases. It is also noteworthy that for *d*_p_ = 238 μm there is a narrower range of bubble diameters around 2 cm, while for the (less well structured) beds of larger particles, the range of bubble sizes is wider. Figure 13 provides a summary of how the relationship between the bubble rising velocity and bubble diameter varies with the pulsation frequency for beds of different particle sizes. For a smaller particle size (Figure 13a), the rising velocity of the bubbles decreases with a reduction in pulsation frequency. As the bubble diameter increases, the effect of lower pulsation frequencies on reducing bubble rise velocity becomes more pronounced. However, as the frequency gradually increases, this difference diminishes. In the case of larger particles (Figure 13b), lower pulsation frequencies still result in slower bubble rising velocities than predicted, but there are no clear effects associated with different pulsation frequencies or bubble diameter. The peaks in Figure 13 for larger bubble diameters at frequencies of 6 and 7 Hz are attributed to the infrequent occurrence of larger bubbles at these frequencies, which are coincidentally captured at or near their peak velocities. Note that the discussion here includes only individual bubbles, not those undergoing coalescence or splitting.

#### 4.2.2 Bubble Size Distribution

**Figure 14:**
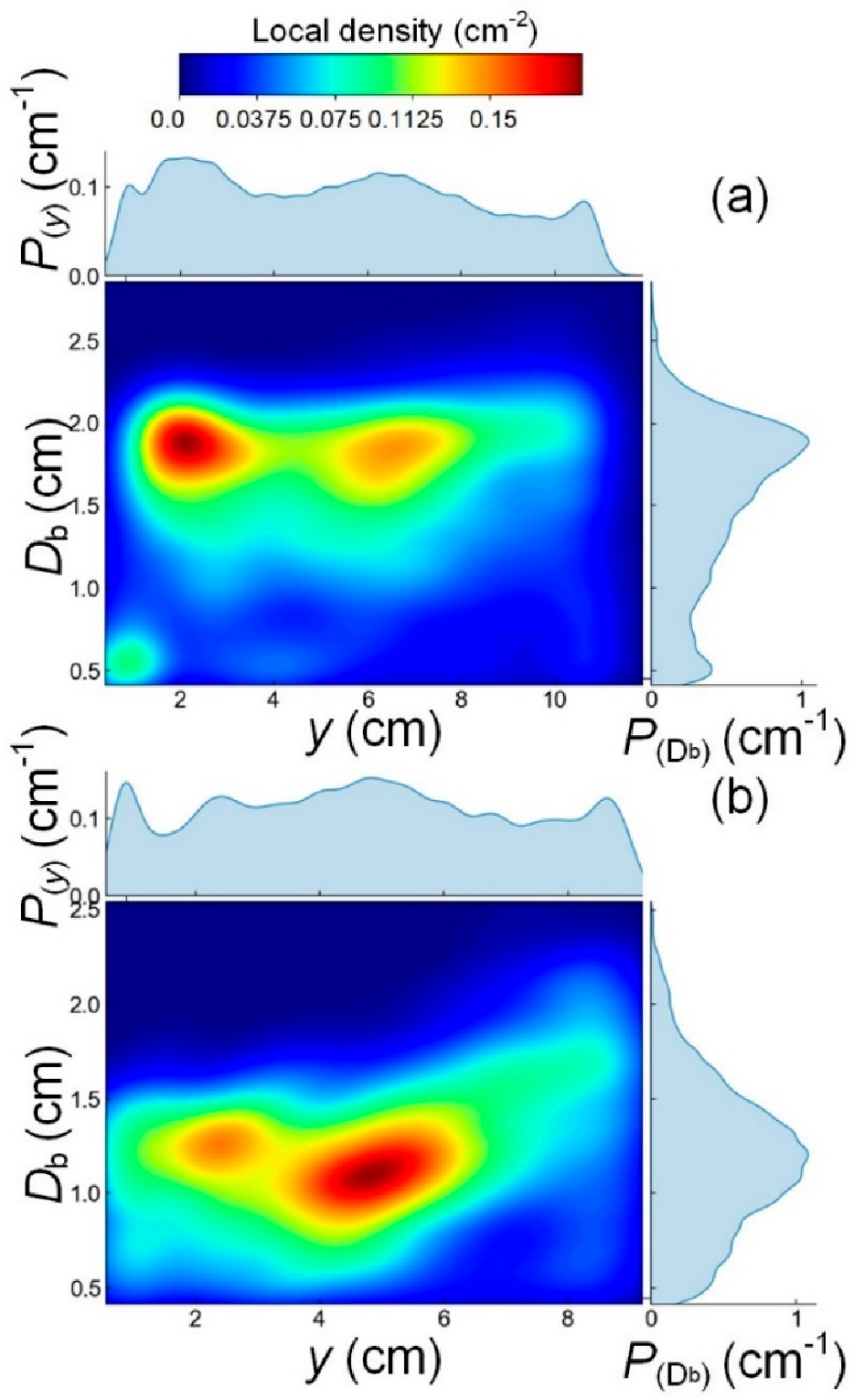
Map of the combined probability density of bubble size and height (*f* = 5 Hz). (a) *d*_p_ = 238 μm,  = 1.50,  = 1.0. (b) *d*_p_ = 550 μm,  = 0.30,  = 0.92.

An accurate segmentation methodology allows for the study of not only bubble size distributions but also subtle changes in the spatial and temporal variation of bubble size. Figure 14 illustrates this capability by displaying the 2D probability density function (PDF) of bubble size and height computed with a kernel density estimate (KDE) and a Gaussian kernel. The frequency represents the most probable combinations of the size and position in the bed. In the case of *d*_p_ = 238 μm, Figure 14a, the size distribution is skewed to the left, and the maximum is close to the gas distributor; in other words, as expected, bubbles are nucleated at a consistent size and position, and do not grow significantly as they rise. A second mode appears at the same size at around 7 cm, likely related to inflation/deflation during pulsation. In contrast, beds of larger particles in Figure 14b show a different dynamic. Bubbles are smaller, but their size distribution not only broadens at the nucleation stage (close to the distributor) but also widens for higher positions, extending beyond 1.5 cm to 2 cm. This more complex behavior results from limited coalescence and breakage. Interestingly, despite the bubble nucleation leading to a less consistent size due to bubble splitting, bubbles rearrange consistently, leading to a strong mode; in other words, all bubbles achieve a consistent size at a higher position in the bed.

#### 4.2.3 Bubble shape

**Figure 15:**
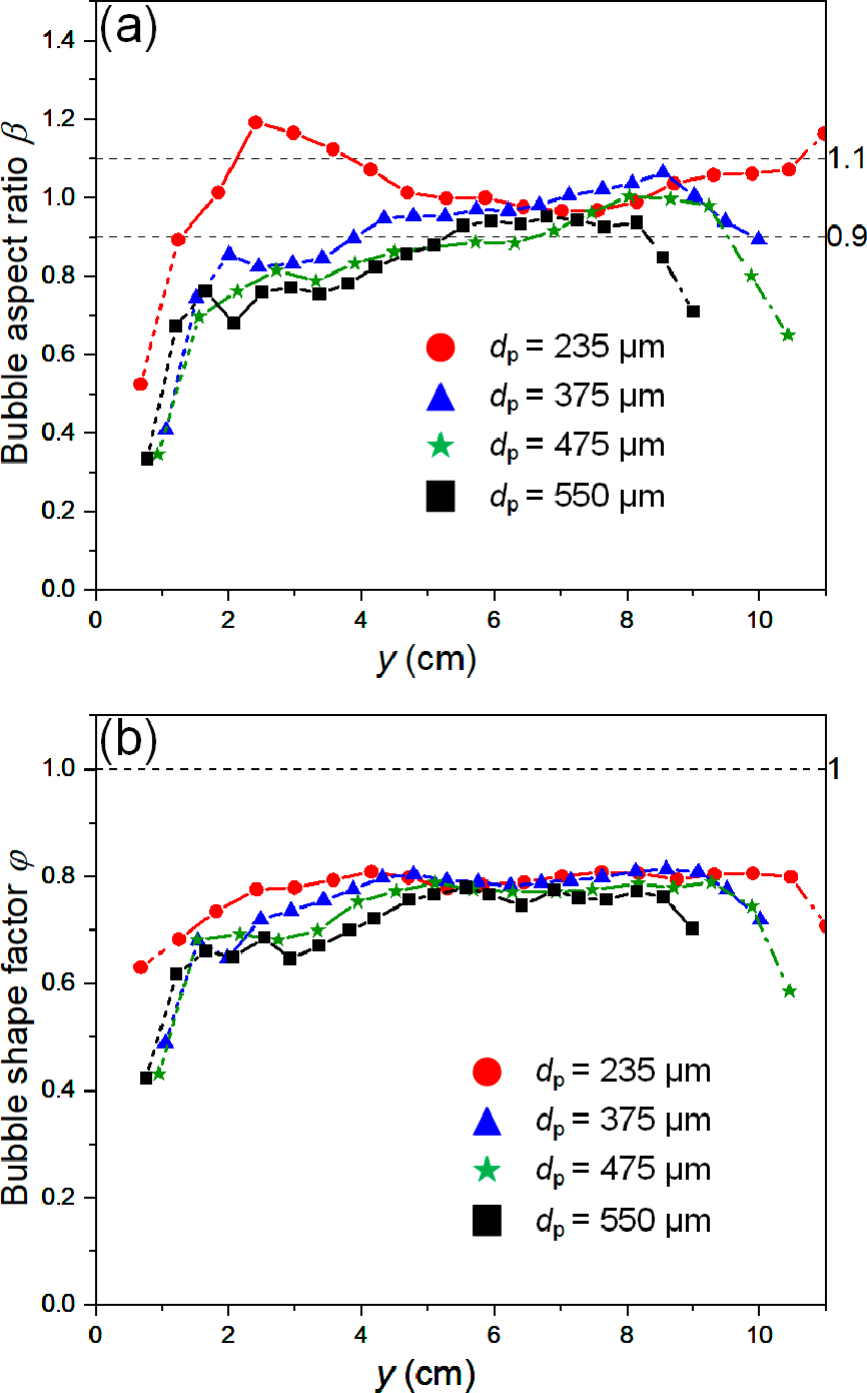
Effects of the bed particle size on the evolution of the (a) bubble aspect ratio and (b) bubble shape factor along the bed height (*f* = 5 Hz). Dash line indicates the nucleation stage, continuous line indicates bubble propagation stage, and dash-dot line indicates rupture stage. Error bars excluded for clarity. Standard error ranged from 0.08 to 0.35.

The interaction between gas and solid particles at the interface of a bubble significantly influences its morphology. Bubbles manifest in various forms: circular (0.9 < β < 1.1), ellipsoidal (β < 0.9), strip-shaped (β ≪ 0.9), or irregular shapes in the pulsed 2D fluidized beds being studied. Figure 15 illustrates how an accurate segmentation method allows for a detailed study of the evolution of bubble morphology, differentiating between different processes. Figure 15a shows small β values near the gas distributor corresponding to the nucleation process, where bubbles appear as horizontal channels. In cases with *d*_p_ = 238 μm, the aspect ratio β increases quickly, suggesting fast nucleation; however, in beds of larger particles, the slope in this region decreases, indicating that nucleation takes a longer distance to complete. During the bubble propagation phase, all cases maintain an aspect ratio between 0.9 and 1.1. However, the stability of circular bubbles is clearly worse in beds of larger particles, where bubbles approaching the bed surface shrink, causing the aspect ratio to drop below 0.9. Figure 15b depicts the corresponding changes of shape factor φ. For all cases, φ tends to concentrate around 0.8 in the main part of the bed, implying that the bubble shape is relatively regular. However, for the nucleation and rupture stages, with an increase in particle size, φ gradually shifts to smaller values, indicating the formation of irregularly shaped bubbles.

#### 4.2.4 Multibubble Dynamics

**Figure 16:**
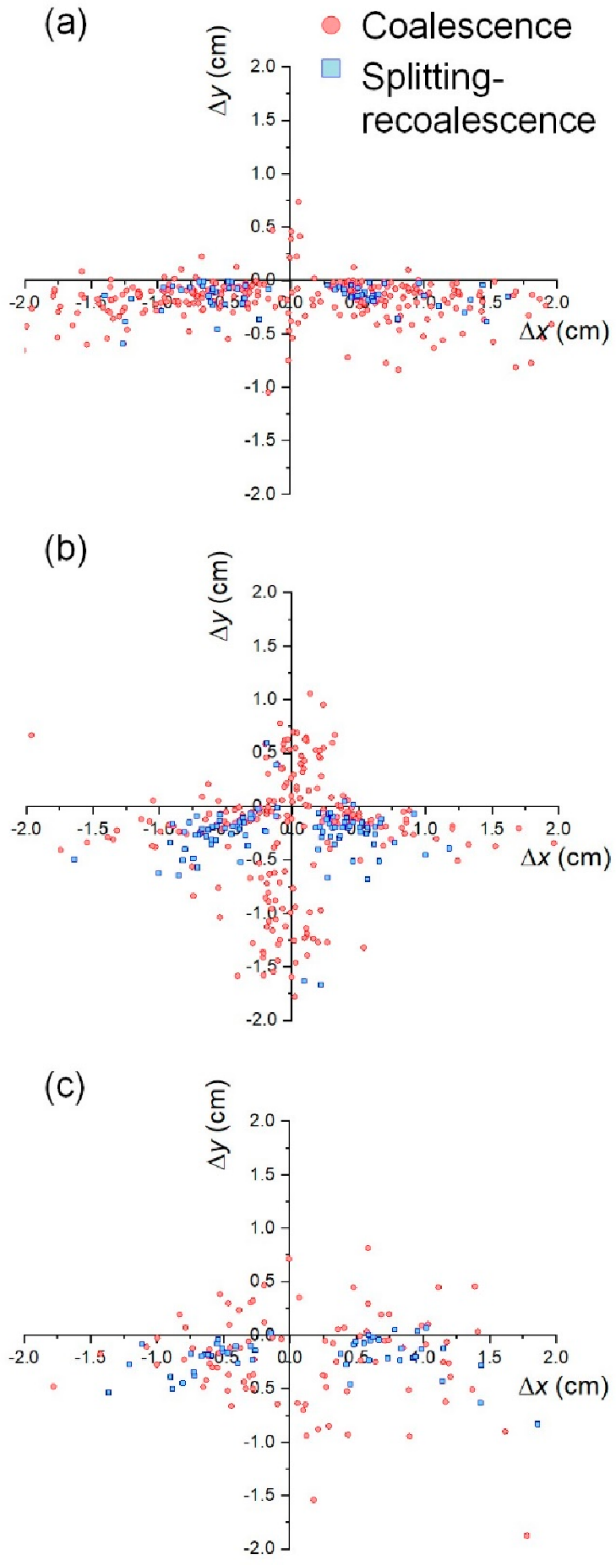
Relative positions of daughter to parent bubbles for different coalescence processes. (a) *d*_p_ = 238 μm, *f* = 3 Hz,  = 1.50,  = 1.0. (b) *d*_p_ = 238 μm, *f* = 7 Hz,  = 1.50,  = 1.0. (c) *d*_p_ = 550 μm, *f* = 5 Hz,  = 0.30,  = 0.92.

The application of the enhanced segmentation method, along with an in-house tracking algorithm, allows quantifying the rate of splitting and coalescence events (see a detailed explanation of the identification methodology in the tracking algorithm in the Supporting Information). Figure 16 shows the resulting spatial distribution of the relative positions between daughter and parent bubbles in bubble coalescence events. Two subgroups are shown: coalescence between two original bubbles and splitting-recoalescence events, in other words, bubbles that are seen to coalesce quickly after having split from the same parent, a phenomenon particularly common in large bubbles and beds of large particles. The analysis provides both a mechanistic understanding and quantification. Figure 16 shows how, in beds of smaller particle size (*d*_p_ = 238 μm) and low pulsation frequency (*f* = 3 Hz), in-plane bubble coalescence occurs, indicated by the purely horizontal relative positions in Figure 16a. However, as the frequency increases (Figure 16b) in-line bubble coalescence becomes more frequent, resulting in common vertical relative positions. When the bed is comprised of larger particles (Figure 16c), the overall number of events drops due to fewer bubbles being formed, and both in-line and in-plane events occur due to the structured flow being less stable (Figure 11) and rendering a more distorted bubble morphology (Figure 15).

**Figure 17:**
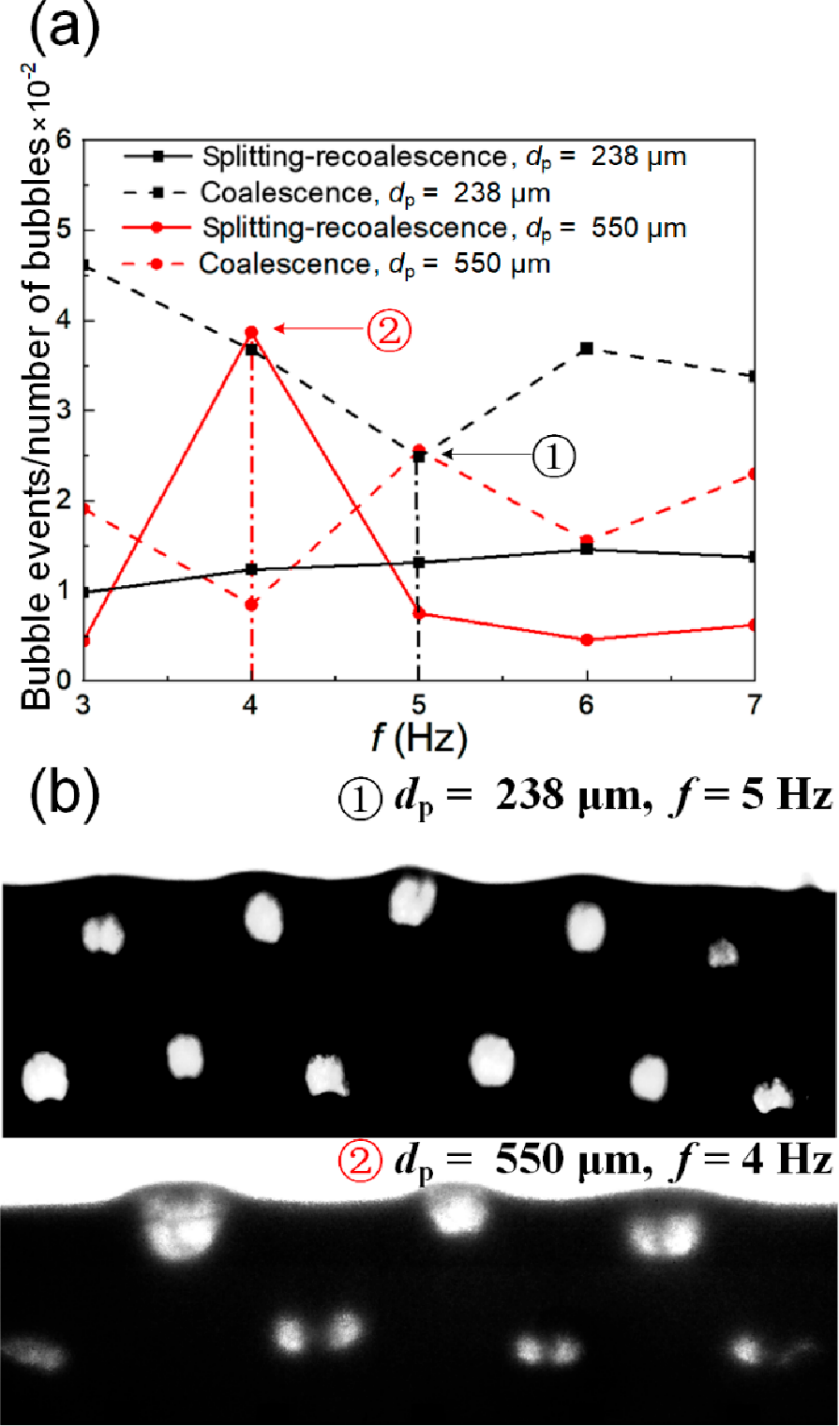
(a) Bubble coalescence relative rate. (b) Corresponding structured bubble images. For *d*_p_ = 238 μm,  = 1.50,  = 1.0, while for *d*_p_ = 550 μm,  = 0.30,  = 0.92.

Figure 17 provides the quantification of these events, reporting the relative rate (the total number of events over the total number of bubbles) for both coalescence and splitting-recoalescence. In beds of smaller particles, the sole-coalescence rates range from 0.025 to 0.046 events per bubble, while quick splitting-recoalescence events account for 21.3 to 52.9% of them. In cases involving larger particles (*d*_p_ = 550 μm), the total number of bubbles is much lower, less than half, resulting in fewer overall events (Figure 16c), as well as an overall drop in the rate (Figure 17a). However, it is interesting to note that at *f* = 4 Hz, the rate of binary bubble splitting-recoalescence events becomes far higher, while sole-coalescence is very low (Figure 17a). Under these conditions, the system is the most well-structured, and bubbles are seen to split and recombine without moving away from their positions in a lattice, as indicated in Figure 17b. This suggests that the compartmentalization of the solid flow, known to stabilize the formation of a bubble pattern, is sufficient to keep daughter bubbles in place and suppress coalescence even when splitting occurs.^51^ Under other conditions, however, when the bed is less well structured, bubbles can move away from their lattice positions and coalesce without recombination, leading to a higher sole-coalescence rate.

## 5 Conclusions

In this work, we have proposed a universally applicable, automated machine-learning-assisted image segmentation method, specifically designed for identifying bubbles in gas–solid fluidized beds. This innovative approach effectively minimizes interference from uneven illumination, internal components, or other obstructive elements within the bed. Combined with binary images segmented by this ML-assisted method, we introduce a Lagrangian tracking technique to capture the evolution of bubble behavior. This technique enables comprehensive analysis of various bubble behaviors, including nucleation, rising, coalescence, splitting, and binary splitting followed by recoalescence.

Applying this method to study the bubble dynamics in a pulsed quasi-2D fluidized bed with Geldart-B particles demonstrates its versatility and effectiveness across different operational conditions and particle sizes. The methodology has shown the ability to differentiate subtle changes in the features of dynamically structured oscillating beds, describing how bubble dynamics, pulsation, and the properties of the solids interplay. As an example, in this work we describe how the structured flow in beds with larger particles is generally more unstable, leading to broader bubble size distributions and increased shape factors resulting from distorted morphological shapes. This, in turn, leads to distinctive mechanisms for bubble coalescence and splitting that can be identified by making use of an event tracking algorithm. While the ML-assisted analysis has proven to be very helpful in obtaining detailed insights into pulsed fluidized bed hydrodynamics, it can clearly be applied more broadly to improve the standardization and transportability of data in the analysis of the hydrodynamics of fluidized beds.

## Supporting Information Available

Additional details on the bubble tracking algorithm and bubble tracking parameters (PDF)
A video demonstrating a comparison of different bubble segmentation methods applied to a conventional bed (Supplementary Video S1) (MP4)
A video demonstrating a comparison of different bubble segmentation methods applied to a dynamically structured pulsed bed with regular bubble patterns (Supplementary Video S2) (MP4)
A video demonstrating a comparison of different bubble segmentation methods applied to a pulsed bed with chaotic bubble patterns (Supplementary Video S3) (MP4)

## Nomenclature

*A*: bubble area, cm^2^
*c*: bubble centroid, cm
*d*_p_: particle diameter, μm
*D*_b_: equivalent bubble diameter, cm
*f*: pulsation frequency, Hz
*H*_0_: initial bed height, cm
*L*: binary cross entropy loss
*k*_1_, *k*_2_: bubble shrinkage and expansion factor
*n*: frame number
*N*_pix_: total number of pixels inside the bubble
*P*_b_: perimeter of the bubble 2D projection, cm
*t*: time, s
*T*: image-independent threshold
*V*_*x*_, *V*_*y*_, *V*: bubble lateral velocity, rising velocity, moving velocity, cm/s
pulsation amplitude
pulsation offset
*U*_mf_: minimum fluidization velocity, cm/s
*y*_i_: pixel *i* in the binary mask image
pixel *i* probability score computed by the sigmoid activation layer
difference of the bubble centroid coordinates in the two consecutive frames, cm
*y*_max_, *x*_max_: maximum length of the bubble in the vertical and horizontal directions, cm

## Greek Letters

α_1_, α_2_: weight parameters
β: bubble aspect ratio
φ: bubble shape factor
ϕ: velocity coefficient
