# Supplementary material for: Machine Learning Assisted Experimental Characterization of Bubble Dynamics in Gas–Solid Fluidized Beds
Source: Ind Eng Chem Res. 2024 May 1;63(19):8819–32. doi: 10.1021/acs.iecr.4c00631 (PMC11099962; doi:10.1021/acs.iecr.4c00631)
Supplement: Supplementary file 1 — ie4c00631_si_001.pdf [file ie4c00631_si_001.pdf]

Supporting Information

Machine learning assisted experimental characterization of bubble dynamics in gas-solid fluidized beds

Shuxian Jiang[a, ‡], Kaiqiao Wu[a, ‡], Victor Francia[b], Yi Ouyang[c], Marc-Olivier Coppens[a,]*

[a] Centre for Nature-Inspired Engineering and Department of Chemical Engineering, University College London, London, WC1E 6BT, United Kingdom

[b] School of Engineering and Physical Sciences, Edinburgh EH14 4AS, Heriot-Watt University, United Kingdom.

[c] Laboratory for Chemical Technology, Ghent University, Ghent 9052, Belgium

*Email addresses: m.coppens@ucl.ac.uk (Marc-Olivier Coppens)

1. Bubble tracking parameters

In the bubble tracking model, k_1 and k_2 are critical parameters. For the validation of the model's robustness and the enhancement of its accuracy, an array of k_1 and k_2 values was examined across various cases. As displayed in Figure S1, the statistical results reveal that the case k_1 = 0.64 and k_2 = 1/k_1 = 1.5625 yields a high rate of correct identifications for bubble

coalescence and splitting events, with a notably low count of incorrect and undermined bubble events. Consequently, these values were chosen for the bubble tracking model to effectively identify and analyze multi-bubble behaviors.

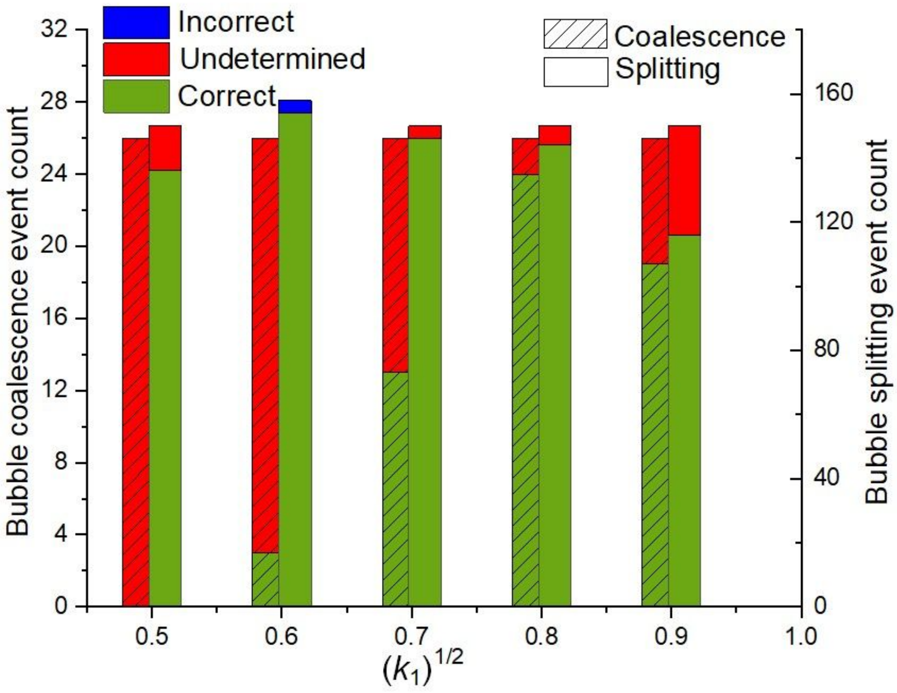

Figure S1. Summary of bubble tracking results with different k_1 and k_2 for bubble coalescence and split-up in pulsed fluidized beds. Input 500 frames were taken from: 125 frames per particle size, distributed across 5 frequency conditions with 25 consecutive frames selected randomly for each.

2. Bubble tracking algorithm

Figure S2 displays the network of bubble tracking, with three rows representing a sequence of bubble track IDs at three successive time frames: n - 1, n, n + 1. This network provides a method for identifying and tracking bubble behaviors between successive time frames. Regarding binary breakup followed by re-coalescence, a different set of rules for bubble tracking is applied.

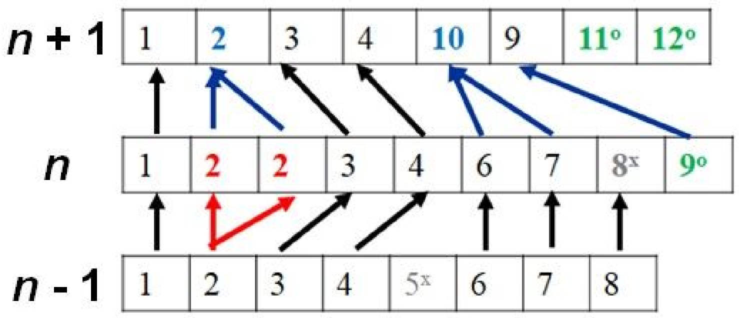

Figure S2. An illustration of the tracking algorithm. Black arrow: bubble rising. Red arrow: bubble splitting. Blue arrow: bubble coalescence.

The arrows among bubble IDs in consecutive frames form a network, illustrating a bubble graph or map. The colors of arrows and bubble IDs signify different types of relationships in the map: black arrows represent bubbles rising, red arrows indicate bubbles splitting, and blue arrows denote bubble coalescence. If the bubble in the current frame is not connected to any earlier bubbles, it means the bubble has just been formed. For the sake of discussion, assume in Figure S2 that $n = 2$. All bubbles in the first frame (n - 1 = 1) are sequentially numbered as, e.g., 1-8, and tagged with a bubble event label [nucleate, rise, coalesce, split] = [1, 0, 0, 0],

along with a velocity matrix [V_x, V_y, V] = [0, 0, 0]. In the second frame, all bubbles are not numbered initially. The algorithm takes the two binary images as inputs. After the algorithm has been executed, the black arrows indicate that bubbles 1, 3, 4, 6, 7, and 8 continue to rise, and so gain a bubble event tag [0, 1, 0, 0]. The red arrows indicate that bubble 2 splits up into two daughter bubbles 2, and so it gains a bubble event tag [0, 0, 0, 1]. For the third frame, the blue arrows indicate that bubbles 6 and 7 coalesce to form a new bubble 10; since the daughter bubbles of 2 have the tag [0, 0, 0, 1], they will keep the bubble ID 2, and an updated bubble event tag [0, 0, 1, 0]. Because bubbles 5 and 8 could not be associated with a bubble in the next frame, their disappearance is associated with a death event ($^\times$), and because bubbles 9, 11, and 12 could not be paired with any bubble in the previous frame, their formation is associated with a birth event ($^\circ$). The full bubble tracking algorithm is presented Table S1.

Figure S3 presents a representative result of tracking bubble dynamics across two pulsation periods, illustrating the algorithm's potential in distinguishing various bubble phenomena throughout the entire bubble lifecycle. For asymmetrical in-plane bubble splitting, smaller bubbles tend to disappear shortly after splitting, as shown in Figure S3, while the larger ones continue to ascend vertically. In the case of symmetrical in-plane bubble splitting, the binary

daughter bubbles often quickly re-coalesce, typically leading to the formation of a new, large bubble.

Table S1. The bubble tracking algorithm.

```
Output: Bubble event tag, properties, and velocities over N snapshots
for bubble i in current frame n (n > 1):
    measure bubble properties for bubble i
    for bubble j in previous frame n-1:
        Retrieve bubble event tag, bubble properties, and velocities for bubble j
        Multiply the pixels of bubble i and bubble j, S
        If S > 0 and condition Eq. (3) is satisfied:
            Assign bubble rising tag [0, 1, 0, 0], calculate velocity using Eq. (7)
            overlap = True
            Break
        else:
            Record the added bubble j into the local overlap bubble list
    If not overlap:
        If local overlap bubble list = 1 and A_{n,i} < A_{n-1, j}:
            Assign bubble event tag [0, 1, 0, 0], calculate velocity using Eq. (7)
        elif count (local overlap bubble list) ≥ 2 and condition Eq. (5) satisfied:
            Assign bubble coalescence tag [0, 0, 1, 0], calculate velocity using Eq. (8)
    else:
        Assign bubble birth tag [1, 0, 0, 0], calculate velocity = [0.0, 0.0, 0.0]
Save bubble event tag, properties, and velocities for frame n
Count bubble ID for all bubbles in frame n:
    if count(bubble ID)> 1:
        Assign bubble splitting tag [0, 0, 0, 1], calculate velocity using Eq. (7)
```

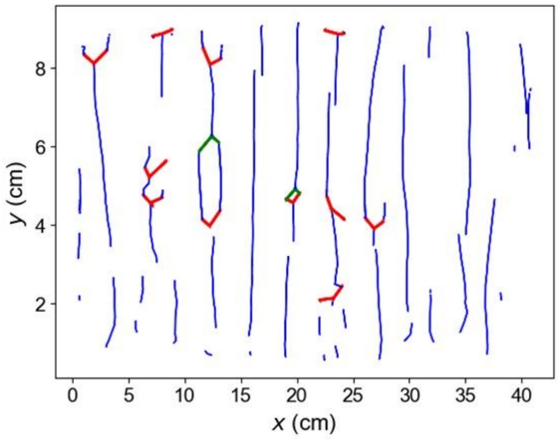

Figure S3. Bubble tracking in pulsed fluidized bed during two periods (t = 0.4 s, f = 5 Hz, d_p = 550 μm, $\hat{\boldsymbol{u}}_{\boldsymbol{a}}$= 0.30, $\hat{\boldsymbol{u}}_{\boldsymbol{min}}$ = 0.92.). Blue line: bubble rising. Red line: bubble splitting. Green line: bubble coalescence.
